# Parental psychological distress during pregnancy and the risk of childhood lower lung function and asthma: a population-based prospective cohort study

**DOI:** 10.1136/thoraxjnl-2019-214099

**Published:** 2020-10-12

**Authors:** Evelien R van Meel, Gautam Saharan, Vincent WV Jaddoe, Johan C de Jongste, Irwin KM Reiss, Henning Tiemeier, Hanan El Marroun, Liesbeth Duijts

**Affiliations:** 1 The Generation R Study Group, Erasmus Medical Center, Rotterdam, The Netherlands; 2 Department of Pediatrics, Division of Respiratory Medicine and Allergology, Erasmus Medical Center, Rotterdam, The Netherlands; 3 Department of Pediatrics, Erasmus Medical Center, Rotterdam, The Netherlands; 4 Department of Pediatrics, Division of Neonatology, Erasmus Medical Center, Rotterdam, Zuid-Holland, The Netherlands; 5 Department of Child and Adolescent Psychiatry, Erasmus Medical Center, Rotterdam, The Netherlands; 6 Department of Epidemiology, Erasmus Medical Center, Rotterdam, The Netherlands; 7 Department of Social and Behavioural Science, Harvard University T H Chan School of Public Health, Boston, Massachusetts, USA; 8 Department of Psychology, Education and Child Studies, Erasmus Universiteit Rotterdam, Rotterdam, The Netherlands

**Keywords:** asthma epidemiology, psychology, paediatric asthma

## Abstract

**Background:**

Although maternal psychological distress during pregnancy is associated with increased risks of respiratory morbidity in preschool children, it is unknown whether this association persists into later childhood.

**Objective:**

To examine the association between parental psychological distress during pregnancy and lung function and asthma in children of school age.

**Methods:**

This study of 4231 children was embedded in a population-based prospective cohort. Parental psychological distress was assessed by the Brief Symptom Inventory during and 3 years after pregnancy, and in mothers also at 2 and 6 months after pregnancy. At age 10 years, lung function was obtained by spirometry and asthma by questionnaire.

**Results:**

The prevalence of asthma was 5.9%. Maternal overall psychological distress during pregnancy was associated with a lower forced vital capacity (FVC) (z-score difference −0.10 (95% CI −0.20 to –0.01) per 1-unit increase), maternal depressive symptoms during pregnancy with a lower forced expiratory volume in the first second (FEV_1_) and FVC (−0.13 (95% CI −0.24 to –0.01) and −0.13 (95% CI −0.24 to –0.02) when using clinical cut-offs) in their children. All maternal psychological distress measures during pregnancy were associated with an increased risk of asthma (range OR: 1.46 (95% CI 1.12 to 1.90) to 1.91 (95% CI 1.26 to 2.91)). Additional adjustment for paternal psychological distress during pregnancy and parental psychological distress after pregnancy did not materially change the associations. Paternal psychological distress during pregnancy was not associated with childhood respiratory morbidity.

**Conclusion:**

Maternal, but not paternal, psychological distress during pregnancy is associated with an increased risk of asthma and partly lower lung function in children. This suggests intrauterine programming for the risk of later-life respiratory disease.

**Key messages**

**What is the key question?**

- Is parental psychological distress during pregnancy associated with lung function and asthma at school age?

**What is the bottom line?**

- Maternal, but not paternal, psychological distress during pregnancy is associated with an increased risk of asthma and partly lower lung function in children.

**Why read on?**

- These findings may indicate an intrauterine effect of maternal psychological distress during pregnancy on fetal lung development and respiratory morbidity rather than an effect of unmeasured genetic, social, behavioural or environmental factors.

## Introduction

Early life is a sensitive period for the development of respiratory health.^1^ We and others have previously shown that maternal psychological distress during pregnancy is associated with increased risks of wheezing and asthma in their preschool-aged children.^2–7^ This suggests a potential role of intrauterine mechanisms, such as altered programming of the fetal hypothalamic–pituitary–adrenal (HPA) axis, leading to adaptive airway and lung development and asthma.^8–10^ The association of maternal psychological distress during pregnancy with childhood asthma might also be explained by residual confounding factors such as unmeasured genetic, social, behavioural or environmental factors. Since these residual confounding factors are shared by mother and father, while only maternal psychological distress might have intrauterine effects, examining paternal psychological distress during pregnancy in relation to childhood asthma is of importance to account for these residual confounders.^3 11^ Only a few studies have measured paternal psychological distress, and they found that this is not associated with an increased risk of respiratory symptoms in early childhood, suggesting an intrauterine effect.^2 3 7^ To date, the associations of maternal and paternal psychological distress during pregnancy with asthma in later childhood and the association with lung function are not fully clear.^12 13^ We hypothesise that maternal psychological distress during pregnancy, but not paternal psychological distress, is associated with lower lung function and asthma in school-age children, suggesting intrauterine programming for the risk of respiratory disease.

We therefore examined the association between maternal psychological distress during pregnancy and lung function and asthma in children aged 10 years in a population-based prospective study. We also assessed whether these associations were independent of paternal psychological distress during pregnancy and parental psychological distress after pregnancy.

## Methods

### Design

This study was embedded in the Generation R Study, a population-based prospective cohort study from early fetal life onwards in Rotterdam, the Netherlands.^14^ The study has been approved by the Medical Ethical Committee of the Erasmus MC, University Medical Centre in Rotterdam. Written informed consent was obtained from all parents or legal guardians of the participants. Children from twin pregnancies (n=185), with missing information on maternal psychological distress during pregnancy (n=1964) or missing information on both lung function and asthma (n=1013) were excluded, which left a total of 4231 subjects at age 10 years for the current analyses (online supplementary figure S1).

10.1136/thoraxjnl-2019-214099.supp1 Supplementary data

#### Maternal and paternal psychological distress

Maternal and paternal psychological distress was assessed by questionnaires completed in the second trimester of pregnancy and 3 years after pregnancy. Both parents were asked to complete their own questionnaires. At 2 and 6 months after pregnancy, only maternal psychological distress was assessed. We used the Brief Symptom Inventory, a validated 53-item self-report questionnaire covering a broad spectrum of psychological distress experienced in the last 7 days.^15 16^ A global scale (Global Severity Index (GSI)) and nine symptom scales were deﬁned.^17 18^ The GSI is a measure of the current level or the depth of symptoms and denotes overall psychological distress. Of the symptom scales, we used the depressive and anxiety symptom scale only since, in general, depression and anxiety are considered the most common psychological distress during and after pregnancy. Other subscales encompass less prevalent disorders such as somatisation, obsessive-compulsion, interpersonal sensitivity, hostility, phobia, paranoia and psychoticism. All scales were repeated at 2 months after pregnancy and, at 6 months and 3 years after pregnancy only depressive and anxiety symptom scales were used. All items (53 for the GSI and 6 for both the depressive and anxiety symptom scales) were rated on a 5-point unidimensional scale ranging from 0 (‘not at all’) to 4 (‘extremely’). Total scores for each scale were calculated by summing the item scores and dividing this by the number of endorsed items. Based on Dutch cut-off values, mothers and fathers were categorised as having clinically relevant psychological distress (no; yes) when having a score of ≥0.71 or ≥0.66 on the global scale, ≥0.80 or ≥0.71 on the depressive symptom scale and ≥0.71 or ≥0.65 on the anxiety symptom scale, respectively.^19^ The Cronbach’s alpha, reflecting internal consistency of the different scales, ranged from 0.72 to 0.93, which is considered acceptable to excellent. Spearman correlations between parental psychological distress during and after pregnancy varied between 0.29 and 0.45 (weak to moderate), and between maternal and paternal psychological distress between 0.12 and 0.23 (very weak to weak).

#### Lung function and asthma in school-age children

Lung function was measured by spirometry at the age of 10 years (median 9.7 (5%–95% range 9.5–10.3)), as reported earlier.^20^ In short, forced expiratory volume in 1 s (FEV_1_), forced vital capacity (FVC), FEV_1_/FVC and forced expiratory flow after exhaling 75% of FVC (FEF_75_) were measured in accordance with the American Thoracic Society (ATS) and European Respiratory Society (ERS) recommendations and converted into sex-, height-, age- and ethnicity-adjusted z-scores according to the Global Lung Initiative reference data.^21 22^ Additionally, we included 281 children whose spirometry did not meet formal reproducibility criteria but who had at least one adequate curve with respect to reach and duration of the plateau according to ATS/ERS criteria. Observed sizes and direction of effect estimates were similar to when these children were excluded.^23^ Current asthma was defined as ever diagnosis of asthma, with either wheezing or any asthma medication use in the past 12 months at the age of 10 years. Information on asthma diagnosis and wheezing was obtained by a parental questionnaire based on the International Study on Asthma and Allergy in Childhood (ISAAC) Questionnaire (“Has your child ever had asthma diagnosed by a doctor?” and “Has your child suffered from attacks of wheezing in the chest in the past 12 months?”).^24^ Information on asthma medication use was obtained during the visit to our research centre.

### Covariates

Information on maternal characteristics included age, parity, ethnicity, educational level, smoking during pregnancy, body mass index at enrolment, history of asthma and atopy and pet keeping, and were obtained from multiple questionnaires during pregnancy. Information on paternal characteristics included age, ethnicity, educational level, smoking before pregnancy, body mass index at enrolment and history of asthma and atopy, and were obtained by a questionnaire during pregnancy. Information on child’s sex, gestational age at birth, and birth weight was obtained from midwife and hospital records. Information on child’s ethnicity was based on questionnaires during pregnancy, and information on breastfeeding and daycare attendance were obtained by questionnaires in the first year of life. Additional information on confounders can be found in the online supplemental methods.

### Statistical analysis

First, we compared the characteristics of children included in and excluded from our study due to loss to follow-up using Mann–Whitney U tests, t-tests and χ^2^ tests. Second, we used linear and logistic regression models to study the associations of maternal psychological distress during pregnancy with lung function and current asthma, respectively. Overall psychological distress, depressive and anxiety symptoms were studied separately, and both continuous measures and clinical cut-offs were used. Analyses were adjusted for confounders, which were first selected from the literature and subsequently included in the model if they were associated with maternal psychological distress and lung function or asthma, or if the effect sizes of unadjusted analyses changed 10% or more after adding a confounder (main model). All confounders selected from the literature met these criteria, and we therefore included all confounders in the model. A directed acyclic graph of the confounders is shown in online supplementary figure S2. Additionally, we added paternal psychological distress during pregnancy to the model to minimise the potential correlated effect of maternal and paternal psychological distress during pregnancy on childhood lung function and asthma, and to assess the individual effect of maternal psychological distress during pregnancy. Next, we added maternal psychological distress after pregnancy to the main model as a mediator to disentangle the individual effect of maternal psychological distress during pregnancy on respiratory morbidity of the child. We assessed the change in effect estimates for the associations of maternal psychological distress during pregnancy with lung function and asthma, after additional adjustment for maternal psychological distress after pregnancy, by using the following formulae for percentage change: 100 * (Effect estimate_full model_ – Effect estimate_original model_)/(Effect estimate_original model_) for continuous lung function measures, and 100 * (Effect estimate_full model_ – Effect estimate_original model_)/(Effect estimate_original model_ – 1) for categorical asthma. All other confounders were kept similar to the model with only maternal distress during pregnancy, to ascertain that the change in effect estimate is only due to the additional adjustment for parental psychological distress after pregnancy.

To assess residual confounding effects of unmeasured genetic, social, behavioural or environmental factors, we studied the associations of paternal psychological distress during pregnancy with lung function and asthma. Additionally, we adjusted the associations for paternal psychological distress after pregnancy including change in effect estimates similar to maternal psychological distress. Since parental psychological distress after pregnancy is likely to be correlated to parental distress during pregnancy, adding psychological distress after pregnancy to the model could lead to multicollinearity. Correlations between parental psychological distress during and after pregnancy were, however, below 0.5. Additionally, we assessed the Variance Inflation Factor and tolerance statistics (which are measures for multicollinearity) of the models where parental psychological distress after pregnancy was added to parental psychological distress during pregnancy. Both these measures showed no indication for multicollinearity and inflation of the model. Lastly, we performed a sensitivity analysis focusing on patterns of depressive and anxiety symptoms. In this analysis, depressive and anxiety symptoms during and after pregnancy were combined into the following groups: never, symptoms during pregnancy only, symptoms after pregnancy only, and both symptoms during and after pregnancy. Symptoms after pregnancy reflect psychological distress at either 2, 6 or 36 months after pregnancy.

Missing data in covariates (range between 0% and 33%), maternal psychological distress after pregnancy and paternal psychological distress during and after pregnancy (range between 26% and 41%) were imputed by multiple imputation using the Markov Chain Monte Carlo method.^25^ We only present the pooled results based on the imputed datasets, since we observed no major differences in the magnitude or direction of the effect when compared with complete case analysis. All measures of association are presented with their 95% CIs. Statistical analyses were performed using SPSS Version 24.0 for Windows software and R version 3.4.1.

## Results

### Characteristics of subjects

Parental and child characteristics are shown in tables 1 and 2, respectively. A total of 362 (8.6%) of the participating mothers and 167 (3.9%) of the participating fathers had clinically relevant overall psychological distress. The mean (SD) values for FEV_1_, FVC, FEV_1_/FVC and FEF_75_ were 2.02 (0.30) L, 2.33 (0.36) L, 86.72 (5.66)% and 1.15 (0.35) L/s, respectively. The prevalence of current asthma in children aged 10 years was 5.9% (n=213). Most prominently, children lost to follow-up more often had non-European parents and a lower gestational age at birth and birth weight (online supplementary table S1).

**Table 1:** Characteristics of mothers and fathers

	n=4231	
	Mothers	Fathers
Mean (SD) age, years	30.9 (4.8)	33.4 (5.6)
Parity, nulliparous	59.5 (2516)	–
Ethnicity, non-European	31.4 (1329)	35.6 (1507)
Education, lower	48.1 (2034)	48.9 (2068)
Smoking during pregnancy, yes	26.5 (1122)	–
Smoking before pregnancy, yes	–	41.6 (1761)
Mean (SD) BMI at enrolment, kg/m^2^	24.5 (4.2)	25.3 (3.4)
History of asthma or atopy, yes	37.7 (1594)	33.7 (1425)
Pet keeping, yes	35.9 (1520)	–
Psychological distress during pregnancy		
Overall psychological distress, continuously*	0.15 (0.00 to 0.91)	0.08 (0.00 to 0.55)
Overall psychological distress, yes	8.6 (362)	3.9 (167)
Depressive symptoms, continuously*	0.00 (0.00 to 1.00)	0.00 (0.00 to 0.67)
Depressive symptoms, yes	8.2 (348)	4.3 (182)
Anxiety symptoms, continuously*	0.17 (0.00 to 1.00)	0.00 (0.00 to 0.75)
Anxiety symptoms, yes	9.3 (395)	8.0 (337)
Psychological distress at 2 months after pregnancy		
Overall psychological distress, continuously*	0.13 (0.00 to 0.93)	–
Overall psychological distress, yes	7.9 (333)	–
Depressive symptoms, continuously*	0.00 (0.00 to 1.08)	–
Depressive symptoms, yes	8.2 (349)	–
Anxiety symptoms, continuously*	0.00 (0.00 to 1.00)	–
Anxiety symptoms, yes	8.3 (350)	–
Psychological distress at 6 months after pregnancy		
Depressive symptoms, continuously*	0.17 (0.00 to 1.17)	–
Depressive symptoms, yes	8.5 (358)	–
Anxiety symptoms, continuously*	0.00 (0.00 to 1.17)	–
Anxiety symptoms, yes	9.9 (417)	–
Psychological distress at 36 months after pregnancy		
Depressive symptoms, continuously*	0.00 (0.00 to 0.78)	0.00 (0.00 to 0.68)
Depressive symptoms, yes	5.1 (217)	4.4 (187)
Anxiety symptoms, continuously*	0.00 (0.00 to 0.80)	0.00 (0.00 to 0.68)
Anxiety symptoms, yes	5.1 (214)	7.7 (324)

Data on maternal depressive (n=5) and anxiety (n=6) symptoms during pregnancy were missing and not imputed.

Values are means (SD),

*medians (5%–95% range), or valid percentages (absolute numbers).

–, not all information was available for both mothers and fathers; BMI, body mass index.

**Table 2:** Characteristics of children

	n=4231
Sex, female	51.2 (2166)
Gestational age at birth, weeks*	40.1 (37.1–42.1)
Birth weight (g)	3450 (545)
Ethnicity, non-European	29.1 (1233)
Ever breastfeeding, yes	90.9 (3847)
Day care attendance first year, yes	57.4 (2428)
FEV_1_, L	2.02 (0.30)
FVC, L	2.33 (0.36)
FEV_1_/FVC, %	86.72 (5.66)
FEF_75,_ L/s	1.15 (0.35)
FEV_1_, z-score	0.17 (0.97)
FVC, z-score	0.21 (0.93)
FEV_1_/FVC, z-score	−0.10 (0.94)
FEF_75,_ z-score	0.04 (0.91)
Current asthma, yes	5.9 (213)

Lung function measures (n=474) and current asthma (n=591) were missing and not imputed.

Values are mean (SD),

*medians (5%–95% range) or valid percentages (absolute numbers).

FEF_75_, forced expiratory flow after exhaling 75% of FVC; FEV_1_, forced expiratory volume in 1 s; FVC, forced vital capacity.

### Maternal psychological distress, lung function and asthma

Unadjusted associations of maternal psychological distress during pregnancy with lung function and asthma in school-age children are shown in online supplementary table S2. After adjustment for confounders and when studied continuously, only maternal overall psychological distress during pregnancy was associated with a lower FVC (z-score difference −0.10 (95% CI −0.20 to –0.01)) in their children (table 3). When using clinical cut-offs, only maternal depressive symptoms during pregnancy were associated with a lower FEV_1_ and FVC (−0.13 (95% CI −0.24 to –0.01) and −0.13 (95% CI −0.24 to –0.02), respectively). Additional adjustment for paternal psychological distress during pregnancy did not change the direction or magnitude of the effect (table 4). When we additionally adjusted for maternal psychological distress at 2, 6 and 36 months after pregnancy and paternal psychological distress 36 months after pregnancy, the size and direction of the associations of depressive symptoms with a lower FEV_1_ and FVC remained unchanged, except when adjusting for maternal psychological distress at 2 months (table 5). The percentage changes in effect estimates were, however, non-significant (data not shown).

**Table 3:** Associations of maternal psychological distress during pregnancy with lung function and asthma in children at age 10 years

	N	FEV_1_ Z-score (95% CI) n=3757	FVC Z-score (95% CI) n=3757	FEV_1_/FVC Z-score (95% CI) n=3757	FEF_75_ Z-score (95% CI) n=3757	Current asthma OR (95% CI) n=3640
Maternal psychological distress						
Overall psychological distress	4231					
Per 1-unit increase		−0.07 (−0.17 to 0.03)	−0.10 (−0.20 to −0.01)*	0.04 (−0.06 to 0.13)	0.01 (−0.08 to 0.10)	1.83 (1.30 to 2.59)**
Clinical cut-off		−0.02 (−0.14 to 0.10)	−0.07 (−0.14 to 0.01)	0.03 (−0.05 to 0.10)	0.02 (−0.05 to 0.09)	1.91 (1.26 to 2.91)**
Depressive symptoms	4225					
Per 1-unit increase		−0.05 (−0.12 to 0.03)	−0.07 (−0.14 to 0.01)	0.03 (−0.04 to 0.11)	0.02 (−0.05 to 0.09)	1.46 (1.12 to 1.90)**
Clinical cut-off		−0.13 (−0.24 to −0.01)*	−0.13 (−0.24 to −0.02)*	−0.01 (−0.12 to 0.11)	−0.03 (−0.14 to 0.08)	1.84 (1.21 to 2.80)**
Anxiety symptoms	4226					
Per 1-unit increase		−0.01 (−0.09 to 0.07)	−0.03 (−0.10 to 0.05)	0.01 (−0.07 to 0.09)	−0.00 (−0.07 to 0.07)	1.55 (1.19 to 2.02)**
Clinical cut-off		−0.01 (−0.12 to 0.10)	0.01 (−0.09 to 0.11)	−0.05 (−0.16 to 0.06)	−0.05 (−0.15 to 0.05)	1.64 (1.09 to 2.47)*

Values are z-scores or odds ratios (OR) with 95% CIs from linear or logistic regression models, respectively.

Maternal psychological distress is treated as a continuous variable (per 1-unit increase) or a dichotomous variable based on clinical cut-offs (no; yes, where ‘no’ was the reference category).

The main models were adjusted for maternal age, parity, education level, smoking during pregnancy, body mass index at enrolment, history of asthma or atopy and pet keeping, and child’s sex, gestational age at birth, birth weight, ethnicity, breastfeeding and daycare attendance.

*P<0.05, **P<0.01.

FEF_75_, forced expiratory flow after exhaling 75% of FVC; FEV_1_, forced expiratory volume in 1 s; FVC, forced vital capacity.

**Table 4:** Associations of maternal psychological distress during pregnancy with lung function and asthma in children at age 10 years, adjusted for paternal psychological distress during pregnancy

	N	FEV_1_ Z-score (95% CI) n=3757	FVC Z-score (95% CI) n=3757	FEV_1_/FVC Z-score (95% CI) n=3757	FEF_75_ Z-score (95% CI) n=3757	Current asthma OR (95% CI) n=3640
Main model + paternal psychological distress						
Overall psychological distress	4231					
Per 1-unit increase		−0.07 (−0.17 to 0.03)	−0.10 (−0.19 to −0.00)*	0.03 (−0.07 to 0.13)	0.01 (−0.09 to 0.10)	1.79 (1.26 2.56)**
Clinical cut-off		−0.02 (-0.14 to 0.10)	−0.05 (−0.16 to 0.06)	0.02 (−0.10 to 0.13)	0.04 (−0.08 to 0.15)	1.92 (1.26 to 2.92)**
Depressive symptoms	4225					
Per 1-unit increase		−0.04 (−0.12 to 0.03)	−0.06 (−0.13 to 0.01)	0.02 (−0.05 to 0.10)	0.02 (−0.05 to 0.09)	1.45 (1.10 to 1.91)**
Clinical cut-off		−0.12 (−0.24 to −0.00)*	−0.12 (−0.23 to −0.01)*	−0.01 (−0.12 to 0.11)	−0.03 (−0.14 to 0.08)	1.82 (1.19 to 2.80)**
Anxiety symptoms	4226					
Per 1-unit increase		−0.01 (−0.09 to 0.07)	−0.02 (−0.10 to 0.05)	0.01 (−0.07 to 0.08)	0.00 (−0.07 to 0.07)	1.57 (1.20 to 2.06)**
Clinical cut-off		0.00 (−0.11 to 0.11)	0.02 (−0.09 to 0.12)	−0.05 (−0.16 to 0.06)	−0.04 (−0.15 to 0.06)	1.64 (1.09 to 2.47)*

Values are z-scores or odds ratios (OR) with 95% CIs from linear or logistic regression models, respectively.

Maternal psychological distress is treated as a continuous variable (per 1-unit increase) or a dichotomous variable based on clinical cut-offs (no; yes, where ‘no’ was the reference category).

The main models were adjusted for maternal age, parity, education level, smoking during pregnancy, body mass index at enrolment, history of asthma or atopy and pet keeping, and child’s sex, gestational age at birth, birth weight, ethnicity, breastfeeding and daycare attendance. Additionally, models were adjusted for paternal psychological distress during pregnancy.

*P<0.05, **P<0.01.

FEF_75_, forced expiratory flow after exhaling 75% of FVC; FEV_1_, forced expiratory volume in 1 s; FVC, forced vital capacity.

**Table 5:** Associations of maternal psychological distress with lung function and asthma in children at age 10 years, adjusted for maternal psychological distress at 2, 6 and 36 months after pregnancy and paternal psychological distress at 36 months after pregnancy

	N	FEV_1_ Z-score (95% CI) n=3757	FVC Z-score (95% CI) n=3757	FEV_1_/FVC Z-score (95% CI) n=3757	FEF_75_ Z-score (95% CI) n=3757	Current asthma OR (95% CI) n=3640
Main model + maternal psychological distress at 2 months						
Overall psychological distress	4231					
Per 1-unit increase		−0.03 (−0.15 to 0.09)	−0.06 (−0.18 to 0.05)	0.05 (−0.08 to 0.17)	0.05 (−0.07 to 0.18)	2.01 (1.26 to 3.22)**
Clinical cut-off		0.01 (−0.12 to 0.14)	−0.02 (−0.15 to 0.10)	0.02 (−0.11 to 0.14)	0.06 (−0.06 to 0.18)	2.11 (1.30 to 3.43)**
Depressive symptoms	4226					
Per 1-unit increase		−0.02 (−0.11 to 0.06)	−0.05 (−0.13 to 0.03)	0.04 (−0.05 to 0.12)	0.04 (−0.04 to 0.13)	1.37 (1.00 to 1.88)
Clinical cut-off		−0.12 (−0.23 to 0.01)	−0.10 (−0.21 to 0.02)	−0.04 (−0.16 to 0.09)	−0.04 (−0.16 to 0.08)	1.87 (1.18 to 2.97)**
Anxiety symptoms	4225					
Per 1-unit increase		0.00 (−0.09 to 0.09)	−0.01 (−0.10 to 0.08)	0.00 (−0.09 to 0.10)	0.00 (−0.09 to 0.09)	1.70 (1.20 to 2.41)**
Clinical cut-off		−0.01 (−0.12 to 0.11)	0.02 (−0.09 to 0.13)	−0.06 (−0.18 to 0.05)	−0.05 (−0.16 to 0.06)	1.72 (1.09 to 2.72)*
Main model + maternal psychological distress at 6 months						
Depressive symptoms	4226					
Per 1-unit increase		−0.05 (−0.14 to 0.04)	−0.06 (−0.14 to 0.03)	0.00 (0.08 to 0.09)	0.01 (−0.08 to 0.09)	1.29 (0.93 to 1.78)
Clinical cut-off		−0.13 (−0.26 to -0.01)*	−0.13 (−0.24 to -0.01)*	−0.02 (−0.15 to 0.10)	−0.04 (−0.16 to 0.08)	1.58 (0.99 to 2.54)
Anxiety symptoms	4225					
Per 1-unit increase		−0.04 (−0.13 to 0.04)	−0.04 (−0.13 to 0.04)	−0.02 (−0.11 to 0.07)	−0.02 (−0.11 to 0.06)	1.61 (1.16 to 2.22)**
Clinical cut-off		−0.02 (−0.13 to 0.09)	−0.01 (−0.11 to 0.12)	−0.07 (−0.18 to 0.05)	−0.06 (−0.17 to 0.05)	1.59 (1.02 to 2.48)*
Main model + maternal psychological distress at 36 months						
Depressive symptoms	4226					
Per 1-unit increase		−0.05 (−0.13 to 0.04)	−0.07 (−0.15 to 0.01)	0.03 (−0.05 to 0.11)	0.03 (−0.06 to 0.11)	1.38 (1.02 to 1.86)*
Clinical cut-off		−0.12 (−0.24 to −0.00)*	−0.12 (−0.24 to −0.01)*	−0.01 (−0.13 to 0.11)	−0.02 (−0.14 to 0.09)	1.78 (1.14 to 2.78)*
Anxiety symptoms	4225					
Per 1-unit increase		−0.03 (−0.11 to 0.06)	−0.03 (−0.12 to 0.05)	−0.00 (−0.09 to 0.08)	−0.01 (−0.09 to 0.07)	1.43 (1.05 to 1.95)*
Clinical cut-off		−0.01 (−0.12 to 0.10)	0.01 (−0.10 to 0.12)	−0.06 (−0.17 to 0.06)	−0.05 (−0.15 to 0.06)	1.52 (0.99 to 2.34)
Main model + paternal psychological distress at 36 months						
Depressive symptoms	4226					
Per 1-unit increase		−0.05 (−0.12 to 0.03)	−0.07 (−0.14 to 0.00)	0.03 (−0.05 to 0.11)	0.02 (−0.05 to 0.10)	1.41 (1.07 to 1.86)*
Clinical cut-off		−0.13 (−0.24 to −0.01)*	−0.13 (−0.24 to −0.02)*	−0.00 (−0.12 to 0.11)	−0.03 (−0.14 to 0.08)	1.78 (1.16 to 2.74)*
Anxiety symptoms	4225					
Per 1-unit increase		−0.02 (−0.09 to 0.06)	−0.03 (−0.11 to 0.04)	0.01 (−0.07 to 0.09)	−0.00 (−0.08 to 0.07)	1.51 (1.15 to 1.99)**
Clinical cut-off		−0.01 (−0.12 to 0.10)	0.01 (−0.10 to 0.11)	−0.05 (−0.16 to 0.06)	−0.05 (−0.15 to 0.05)	1.60 (1.06 to 2.41)*

Values are z-scores or odds ratios (OR) with 95% CIs from linear or logistic regression models, respectively.

Maternal psychological distress is treated as a continuous variable (per 1-unit increase) or a dichotomous variable based on clinical cut-offs (no; yes, where ‘no’ was the reference category).

The main models were adjusted for maternal age, parity, education level, smoking during pregnancy, body mass index at enrolment, history of asthma or atopy and pet keeping, and child’s sex, gestational age at birth, birth weight, ethnicity, breastfeeding and daycare attendance. Additionally, models were adjusted for maternal psychological distress at 2, 6 or 36 months after pregnancy or for paternal psychological distress at 36 months after pregnancy. At 6 and 36 months after pregnancy, not all subscales were measured and therefore overall psychological distress could not be included at these time points.

*P<0.05, **P<0.01.

FEF_75_, forced expiratory flow after exhaling 75% of FVC; FEV_1_, forced expiratory volume in 1 s; FVC, forced vital capacity.

Maternal overall psychological distress and depressive and anxiety symptoms during pregnancy were all associated with an increased risk of current asthma in their children, both continuously and when using clinical cut-offs (range OR 1.46 (95% CI 1.12 to 1.90) to 1.91 (95% CI 1.26 to 2.91)) (table 3). When we additionally adjusted for paternal psychological distress during pregnancy, the associations of maternal psychological distress with childhood asthma remained essentially unchanged (table 4). Additional adjustment for maternal psychological distress at 2, 6 and 36 months after pregnancy did not change the direction or magnitude of the effect estimates for the association with asthma, including a non-significant percentage change in effect estimates (table 5). Analysis of patterns of psychological distress showed that mostly depressive or anxiety symptoms both during and after pregnancy are associated with an increased risk of asthma in children (online supplementary table S3). Separating the confounders into three different groups, including lifestyle and health-related factors, socioeconomic factors and birth and early childhood factors, showed no differences in size or direction of effect estimates of the associations of maternal psychological distress with asthma in their children (online supplementary table S4). However, lifestyle and health-related factors seemed to account for attenuation of the effect of maternal psychological distress with FEF_75_ only.

### Paternal psychological distress, lung function and asthma

Paternal psychological distress during pregnancy was not associated with lung function or asthma in their children (table 6). The associations of paternal psychological distress during pregnancy with lung function and asthma remained essentially unchanged after additional adjustment for paternal psychological distress at 36 months after pregnancy (online supplementary table S5). Only the associations of clinical cut-offs of anxiety with FEV_1_ and FVC became significant, as opposed to before adjustment for paternal psychological distress after birth (−0.15 (95% CI −0.29 to –0.00) and −0.14 (95% CI −0.29 to –0.00), respectively). However, the percentage changes for these and other associations, when adding paternal psychological distress after pregnancy to the model, were non-significant (online supplementary table S6).

**Table 6:** Associations of paternal psychological distress with lung function and asthma in children at age 10 years

	N	FEV_1_ Z-score (95% CI) n=3757	FVC Z-score (95% CI) n=3757	FEV_1_/FVC Z-score (95% CI) n=3757	FEF_75_ Z-score (95% CI) n=3757	Current asthma OR (95% CI) n=3640
Paternal psychological distress						
Overall psychological distress	4231					
Per 1-unit increase		0.00 (−0.18 to 0.18)	−0.05 (−0.23 to 0.13)	0.07 (−0.08 to 0.22)	0.03 (−0.13 to 0.19)	1.13 (0.56 to 2.26)
Clinical cut-off		−0.05 (−0.30 to 0.20)	−0.08 (−0.31 to 0.14)	0.04 (−0.17 to 0.24)	−0.04 (−0.25 to 0.17)	0.98 (0.44 to 2.18)
Depressive symptoms	4231					
Per 1-unit increase		−0.02 (−0.18 to 0.14)	−0.04 (−0.19 to 0.11)	0.02 (−0.10 to 0.14)	−0.01 (−0.16 to 0.13)	1.04 (0.59 to 1.85)
Clinical cut-off		−0.07 (−0.30 to 0.16)	−0.07 (−0.30 to 0.16)	−0.02 (−0.20 to 0.17)	−0.06 (−0.25 to 0.13)	1.04 (0.43 to 2.53)
Anxiety symptoms	4231					
Per 1-unit increase		−0.01 (−0.17 to 0.15)	−0.03 (−0.19 to 0.14)	0.02 (−0.10 to 0.14)	−0.01 (−0.14 to 0.13)	0.85 (0.46 to 1.58)
Clinical cut-off		−0.13 (−0.28 to 0.02)	−0.13 (−0.27 to 0.01)	0.01 (−0.14 to 0.15)	−0.06 (−0.21 to 0.09)	0.99 (0.51 to 1.92)

Values are z-scores or odds ratios (OR) with 95% CIs from linear or logistic regression models, respectively.

Paternal psychological distress is treated as a continuous variable (per 1-unit increase) or a dichotomous variable based on clinical cut-offs (no; yes, where ‘no’ was the reference category).

The models were adjusted for maternal age, parity, education level, smoking during pregnancy, body mass index at enrolment, history of asthma or atopy and pet keeping, and child’s sex, gestational age at birth, birth weight, ethnicity, breastfeeding and daycare attendance and maternal psychological distress during pregnancy.

FEF_75_, forced expiratory flow after exhaling 75% of FVC; FEV_1_, forced expiratory volume in 1 s; FVC, forced vital capacity.

## Discussion

The results of this population-based prospective cohort study showed that children of mothers with overall psychological distress and depressive or anxiety symptoms during pregnancy had an increased risk of asthma at the age of 10 years. Children of mothers with overall psychological distress when measured continuously had a lower FVC, and children of mothers with depressive symptoms when using clinical cut-offs had a lower FEV_1_ and FVC. These findings were mostly independent of paternal psychological distress during pregnancy, and maternal and paternal psychological distress after pregnancy. The strongest effects were found for mothers who experienced psychological distress both during and after pregnancy. Although the effects of distress during pregnancy only were not significant, this is most likely due to the power of the study, given that the effects estimates are in the same range as for the main analysis. Paternal psychological distress during pregnancy was not associated with an increased risk of lower lung function or asthma. Our results may indicate an intrauterine effect of maternal psychological distress during pregnancy on fetal lung development and respiratory morbidity rather than an effect of unmeasured genetic, social, behavioural or environmental factors.

### Comparison with previous studies

Studies examining the association of maternal psychological distress with childhood lung function are scarce. One study found that high prenatal but also postnatal maternal psychological distress, measured as negative life events, was associated with a lower FEV_1_, FVC and mid-expiratory flow (FEF_25-75_) but not FEV_1_/FVC at age 7 years.^26^ This is in line with our study, where we found that the association of maternal psychological distress with a lower FEV_1_ and FVC remains present in later childhood. These significant findings, compared with the more robust findings for the asthma outcome, are few and effect sizes are small from a clinical perspective but of importance from an aetiological perspective. Studies examining the association of maternal psychological stress during pregnancy with wheezing and asthma in children have been summarised in two recent meta-analyses.^5 6^ Both meta-analyses focused mostly on wheezing and asthma until preschool age, with only a few studies that measured respiratory outcomes after the age of 6 years. One meta-analysis including 10 studies found that maternal psychological stress during pregnancy was associated with a 1.56-fold increased risk of any respiratory morbidity in the children.^5^ When the associations with asthma and wheezing were studied separately, the effect sizes were approximately in the same range, although the latter showed high heterogeneity. The other more recent meta-analysis including 24 studies demonstrated that any maternal psychological stress during pregnancy was associated with a 1.13-fold increased risk of asthma in the children.^6^ This was most likely driven by anxiety during pregnancy, since only this association was significant when different types of psychological distress were studied separately. Anxiety, depression and negative life events during pregnancy were all associated with an increased risk of wheezing (OR or RR 1.19, 1.74 and 1.23, respectively). Our study shows that maternal stress during pregnancy is associated with an increased risk of asthma even at a later age, with effect sizes within the same range. Additionally, we demonstrated that these results are independent of paternal psychological distress during pregnancy and parental psychological distress after pregnancy, which was not studied in these meta-analyses.

### Possible mechanisms

Our results suggest that intrauterine mechanisms may underlie the associations of maternal psychological distress with childhood lung function and asthma in their children, rather than unmeasured genetic, social, behavioural or environmental factors. One potential intrauterine mechanism is excess glucocorticoid production due to maternal psychological distress, which could lead to impaired development of the fetal HPA axis.^8^ Additionally, maternal psychological distress could influence the stimulation of corticotrophin releasing hormone (CRH) secretion, which results in increased CRH levels in the fetal circulation and could overstimulate the fetal HPA axis. Moreover, glucocorticoid-regulated genes are key to fetal lung development, especially during the first and second trimesters of pregnancy.^9^ Any disruption in this process could lead to developmental adaptations of the lungs and, hence, altered lung function.

Epigenetics has also been proposed as a possible mechanism. It has been shown that maternal depression and anxiety are associated with methylation of the glucocorticoid receptor gene NR3C1 in cord blood.^27^ Additionally, one study demonstrated an association of prenatal maternal stress with differently methylated regions related to the HPA axis, immune responses and lung organogenesis.^28^

Finally, oxidative stress and the microbiome have been speculated as possible underlying mechanisms for the association of maternal psychological distress during pregnancy with lung function and asthma in their offspring.^29–31^ However, these mechanisms should be studied in more detail.

### Strengths and limitations of the study

The major strength of this study is the use of a large population-based prospective cohort with follow-up until school age. In our study, we used a well-known measure of psychological distress frequently used in epidemiological studies to assess psychological distress at multiple time points, both continuously and with clinical cut-offs. Additionally, we adjusted for multiple possible confounding factors and considered paternal psychological distress to assess unmeasured confounding factors. Some methodological limitations should be discussed. As in any prospective cohort study, our population was subject to loss to follow-up. Of those with singleton liveborn children with consent at age 10 years, 73% responded to the questionnaire measuring psychological distress. This loss to follow-up could lead to bias if the associations of maternal psychological distress during pregnancy with lung function and asthma were different between those included and those not included in the study. This could lead to both an underestimation or an overestimation of the association and, although this is unlikely, it cannot be excluded.^32^ Additionally, self-reporting of exposure and outcomes could potentially lead to information bias which could result in misclassification. The use of validated questionnaires could have minimised the information bias. The prevalence of asthma seems relatively low, but lies within national and worldwide prevalence rates.^33 34^ We consider self-reported asthma as truly asthma because questions were based on the validated ISAAC questionnaire, and wheezing or asthma medication use in the past 12 months was used to better define current asthma. Also, the results were similar when we used persistent asthma as an outcome (data not shown). Moreover, psychological distress was measured only once during pregnancy. Hence, we do not know whether maternal psychological distress varied in intensity or persistence throughout pregnancy. However, one study that measured maternal anxiety at two time points during pregnancy demonstrated associations with asthma that were comparable for both time points, suggesting that timing in pregnancy seems to have a less prominent role in prenatal programming.^2^ Further studies are needed to confirm these results. Lastly, although we adjusted for numerous confounders, we might not have had information on all possible confounding factors such as maternal or child genetic predisposition for psychological stress disorders or childhood exposures such as air pollution. Although some of their effects might be minimal and are highly correlated with presently used confounders, they could potentially have a substantial effect given the relatively small prevalence of asthma.

## Conclusion

Our results suggest a possible intrauterine effect of maternal psychological distress during pregnancy on the risk of asthma and partly lower lung function in children at the age of 10 years. The results were independent of maternal psychological distress after pregnancy and paternal psychological distress during pregnancy and after pregnancy. Further studies are needed to explore underlying mechanisms.
