# Supplementary material for: Parental psychological distress during pregnancy and the risk of childhood lower lung function and asthma: a population-based prospective cohort study
Source: Thorax. 2020 Oct 12;75(12):1074–81. doi: 10.1136/thoraxjnl-2019-214099 (PMC7677473; doi:10.1136/thoraxjnl-2019-214099)

Supplementary material

Parental psychological distress during pregnancy and the risk of childhood lower lung function and asthma: a population-based prospective cohort study.

Evelien R. van Meel, MD*[1,2], Gautam Saharan, MD*[1,2], Vincent W.V. Jaddoe, MD PhD[1,3], Johan C. de Jongste, MD PhD[2], Irwin K. Reiss, MD PhD[4], Henning Tiemeier, MD PhD[5,6,7], Hanan El Marroun PhD[3,5,8], Liesbeth Duijts, MD PhD[2,4]

[1]The Generation R Study Group; Erasmus MC University Medical Center, [2]Department of Pediatrics, Division of Respiratory Medicine; Erasmus MC University Medical Center, [3]Department of Pediatrics; Erasmus MC University Medical Center, [4]Department of Pediatrics, Division of Neonatology; Erasmus MC University Medical Center, [5]Department of Child and Adolescent Psychiatry; Erasmus MC University Medical Center Rotterdam, [6]Department of Epidemiology; Erasmus MC University Medical Center, [7]Department of Social and Behavioural Science; Harvard TH Chan School of Public Health, [8]Department of Psychology, Education and Child Studies; Erasmus School of Social and Behavioral Sciences, Erasmus University Rotterdam * Both authors equally contributed.

Supplemental text

Supplementary Table S1. Subject characteristics for those included and not included in the study.

Supplementary Table S2. Unadjusted associations of maternal psychological distress during pregnancy with lung function and asthma at age 10 years

Supplementary Table S3. Associations of patterns of parental psychological distress with lung function and asthma at age 10 years.

Supplementary Table S4. Association of maternal psychological distress with lung function and asthma at age 10 years, adjusted for different groups of confounders.

Supplementary Table S5. Associations of paternal psychological distress during pregnancy with lung function and asthma at age of 10 years, adjusted for paternal psychological distress at 36 months after pregnancy.

van Meel ER, *et al. Thorax* 2020; 75:1074–1081. doi: 10.1136/thoraxjnl-2019-214099

Supplementary Table S6. Percentage change for associations of paternal psychological distress during pregnancy with lung function and asthma at age 10 years, adjusted for paternal psychological distress at 36 months after pregnancy.

Supplementary Figure S1. Flowchart of participants included for analysis

Supplementary Figure S2. Directed acyclic graph

van Meel ER, *et al. Thorax* 2020; 75:1074–1081. doi: 10.1136/thoraxjnl-2019-214099

Supplemental text

Covariates Information on maternal characteristics included age (years), parity (nulliparous; multiparous), ethnicity (European; non-European), educational level (low-middle; high), smoking during pregnancy (yes; no), body mass index at enrolment (kg/m^2), history of asthma and atopy (yes; no), and pet keeping (yes; no), and were obtained from multiple questionnaires during pregnancy. Information on paternal characteristics included age (years), ethnicity (European; non-European), educational level (low-middle; high), smoking before pregnancy (yes; no), body mass index at enrolment (kg/m^2), and history of asthma and atopy (yes; no), and were obtained by a questionnaire during pregnancy. Information on child's sex (female; male), gestational age at birth (weeks), and birthweight (grams) were obtained from midwife and hospital records. Information on child's ethnicity (European; non-European) was based on questionnaires during pregnancy, and information on breastfeeding (yes; no) and daycare attendance (yes; no) were obtained by questionnaires in the first year of life. The main models were adjusted for maternal age, parity, educational level, smoking during pregnancy, body mass index at enrolment, history of asthma or atopy and pet keeping, and child's sex, gestational age at birth, birthweight, ethnicity, breastfeeding and daycare attendance.

van Meel ER, *et al. Thorax* 2020; 75:1074–1081. doi: 10.1136/thoraxjnl-2019-214099

Supplementary Table S1. Subject characteristics for those included and not included in the study.

	**Included** n = 4,231	**Not included** n = 3,162	**P-value for difference**
**Maternal characteristics**			
Age (years)	30.9 (4.8)	30.1 (7.8)	**<0.001**
Parity, nulliparous (%)	59.5 (2,516)	47.1 (1,488)	**<0.001**
Ethnicity, non-European (%)	30.9 (1,289)	45.1 (1,325)	**<0.001**
Education, lower (%)	47.2 (1,937)	60.8 (1,587)	**<0.001**
Smoking during pregnancy, yes (%)	24.2 (941)	22.2 (366)	0.112
History of asthma or atopy, yes (%)	37.6 (1,434)	36.2 (591)	0.329
Pet keeping, yes (%)	34.9 (1,310)	29.6 (348)	**0.001**
Body mass index at enrolment (kg/m^2)	24.5 (4.17)	25.2 (4.7)	**<0.001**
Maternal overall psychological distress during pregnancy[1]	0.15 (0.00, 0.91)	0.15 (0.00, 1.21)	0.268
**Paternal Characteristics**			
Age (years)	33.4 (5.4)	33.3 (5.5)	0.790
Ethnicity, non-European (%)	25.1 (865)	34.5 (496)	**<0.001**
Education, lower (%)	54.7 (1,743)	49.6 (598)	**0.003**
Smoking before pregnancy, yes (%)	42.2 (1,639)	42.5 (517)	0.307
History of asthma or atopy, yes (%)	32.2 (1,005)	32.6 (360)	0.803
Body mass index at enrollment (kg/m^2)	25.2 (3.3)	25.4 (3.6)	0.070
Paternal overall psychological distress during pregnancy[1]	0.06 (0.00, 0.51)	0.08 (0.00, 0.54)	0.568
**Child characteristics**			
Sex, female (%)	51.2 (2,166)	48.1 (1,520)	**0.008**
Gestational age at birth (weeks)[1]	40.1 (37.1, 42.1)	39.9 (36.0, 42.0)	**<0.001**
Birth weight (grams)	3,450 (544)	3,354 (605)	**<0.001**
Ethnicity, non-European *(%)*	28.7 (1,207)	41.5 (1,220)	**<0.001**
Ever breastfeeding, yes (%)	92.6 (3,290)	91.7 (1,390)	0.252
Day care attendance 1st year, yes (%)	63.3 (1,791)	58.1 (548)	**0.005**

Values are means (SD), [1]medians (5-95% range) or valid percentages (absolute numbers) based on observed data. P values for difference are calculated by independent sample T-test for continuous variables with a normal distribution, the Mann-Whitney U-test for continuous variables with a skewed distribution, and Pearson's Chi-square test for categorical variables.

van Meel ER, *et al. Thorax* 2020; 75:1074–1081. doi: 10.1136/thoraxjnl-2019-214099

Supplementary Table S2. Unadjusted associations of maternal psychological distress during pregnancy with lung function and asthma at age 10 years

	n	FEV_1 Z-score (95% CI) n = 3,757	FVC Z-score (95% CI) n = 3,757	FEV_1/FVC Z-score (95% CI) n = 3,757	FEF_{75} Z-score (95% CI) n = 3,757	Current asthma OR (95% CI) n = 3,640
**Maternal psychological distress**						
**Overall psychological distress**	4,231					
Per 1-unit increase		0.07 (-0.03, 0.16)	0.03 (-0.06, 0.12)	0.07 (-0.02, 0.16)	**0.16 (0.07, 0.24)****	**2.47 (1.82, 3.34)****
Clinical cut-off		0.010 (-0.01, 0.22)	0.06 (-0.05, 0.17)	0.06 (-0.05, 0.17)	**0.18 (0.07, 0.29)****	**2.78 (1.89, 4.07)****
**Depressive symptoms**	4,225					
Per 1-unit increase		0.05 (-0.02, 0.12)	0.20 (-0.05, 0.09)	0.06 (-0.02, 0.13)	**0.12 (0.05, 0.19)****	**1.79 (1.41, 2.26)****
Clinical cut-off		-0.01 (-0.12, 0.10)	-0.02 (-0.13, 0.09)	0.03 (-0.08, 0.14)	0.10 (-0.01, 0.21)	**2.58 (1.74, 3.83)****
**Anxiety symptoms**	4,226					
Per 1-unit increase		0.06 (-0.01, 0.14)	0.05 (-0.03, 0.12)	0.02 (-0.05, 0.10)	**0.08 (0.01, 0.15)***	**1.92 (1.50, 2.46)****
Clinical cut-off		0.08 (-0.03, 0.18)	0.09 (-0.01, 0.19)	-0.03 (-0.13, 0.08)	0.05 (-0.05, 0.15)	**2.18 (1.48, 3.22)****

Values are Z-scores or odds ratios (OR) with 95% confidence interval (95% CI) from linear or logistic regression models, respectively. Maternal psychological distress is treated as continuous variables (per 1-unit increase) or dichotomous variables based on clinical cut-offs (no; yes, where 'no' was the reference category). Forced Expiratory Flow in 1 second (FEV_1), Forced Vital Capacity (FVC), Forced Expiratory Flow after exhaling 95% of FVC (FEF_{75}).
. *p-value <0.05, **p-value <0.01.

van Meel ER, *et al. Thorax* 2020; 75:1074–1081. doi: 10.1136/thoraxjnl-2019-214099

Supplementary Table S3. Association of patterns of parental psychological distress with lung function and asthma at age 10 years.

	n	FEV_1 Z-score (95% CI) n = 3,757	FVC Z-score (95% CI) n = 3,757	FEV_1/FVC Z-score (95% CI) n = 3,757	FEF_{75} Z-score (95% CI) n = 3,757	Current asthma OR (95% CI) n = 3,640
**Maternal psychological distress**						
**Depressive symptoms patterns**						
Never	3,457	Reference	Reference	Reference	Reference	Reference
Prenatal only	142	-0.15 (-0.35, 0.04)	-0.13 (-0.31, 0.07)	-0.05 (-0.24, 0.14)	-0.04 (-0.22, 0.14)	1.31 (0.61, 2.79)
Postnatal only	421	-0.03 (-0.18, 0.11)	-0.06 (-0.18, 0.06)	0.05 (-0.08, 0.17)	-0.01 (-0.15, 0.13)	1.00 (0.55, 1.83)
Both pre- and postnatal	206	-0.09 (-0.25, 0.07)	-0.12 (-0.27, 0.04)	0.03 (-0.12, 0.18)	-0.02 (-0.17, 0.13)	**2.20 (1.31, 3.70)****
**Anxiety symptoms patterns**						
Never	3,399	Reference	Reference	Reference	Reference	Reference
Prenatal only	161	-0.01 (-0.18, 0.17)	-0.00 (-0.19, 0.18)	-0.01 (-0.20, 0.18)	0.01 (-0.16, 0.18)	1.28 (0.62, 2.65)
Postnatal only	431	0.00 (-0.13, 0.14)	-0.03 (-0.15, 0.09)	0.06 (-0.06, 0.17)	0.02 (-0.10, 0.14)	1.08 (0.66, 1.76)
Both pre- and postnatal	234	-0.01 (-0.15, 0.14)	0.02 (-0.13, 0.16)	0.07 (-0.23, 0.08)	-0.09 (-0.23, 0.05)	**1.92 (1.16, 3.20)***
**Paternal psychological distress[1]**						
**Depressive symptoms patterns**						
Never	3,907	Reference	Reference	Reference	Reference	Reference
Prenatal only	136	-0.06 (-0.33, 0.22)	-0.05 (-0.31, 0.22)	-0.04 (-0.26, 0.17)	-0.07 (-0.31, 0.18)	0.91 (0.28, 3.03)
Postnatal only	142	0.07 (-0.14, 0.27)	0.10 (-0.12, 0.32)	-0.08 (-0.32, 0.16)	-0.09 (-0.33, 0.14)	1.26 (0.50, 3.18)
Both pre- and postnatal	46	-0.13 (-0.48, 0.22)	-0.18 (-0.52, 0.16)	0.06 (-0.31, 0.43)	-0.05 (-0.41, 0.30)	1.28 (0.21, 7.84)
**Anxiety symptoms patterns**						
Never	3,666	Reference	Reference	Reference	Reference	Reference
Prenatal only	241	-0.15 (-0.31, 0.00)	-0.14 (-0.29, 0.01)	-0.02 (-0.20, 0.16)	-0.09 (-0.28, 0.09)	1.09 (0.52, 2.28)
Postnatal only	228	0.06 (-0.14, 0.27)	0.08 (-0.10, 0.26)	-0.02 (-0.18, 0.13)	0.01 (-0.14, 0.16)	1.44 (0.81, 2.57)
Both pre- and postnatal	96	-0.06 (-0.33, 0.22)	-0.09 (-0.33, 0.15)	0.07 (-0.19, 0.33)	0.03 (-0.25, 0.31)	0.82, 0.25, 2.70)

Values are Z-scores or odds ratios (OR) with 95% confidence interval (95% CI) from linear or logistic regression models, respectively. Parental psychological distress is treated as continuous variables (per 1-unit increase) or dichotomous variables based on clinical cut-offs (no; yes, where 'no' was the reference category). Postnatal distress reflect psychological distress at either 2, 6 or 36 months after pregnancy. Forced Expiratory Flow in 1 second (FEV_1), Forced Vital Capacity (FVC), Forced Expiratory Flow after exhaling 95% of FVC (FEF_{75}). The models were adjusted for maternal age, parity, education level, smoking during pregnancy, body mass index at enrolment, history of asthma or atopy and pet keeping, and child's

BMJ Publishing Group Limited (BMJ) disclaims all liability and responsibility arising from any reliance placed on this supplemental material which has been supplied by the author(s)

sex, gestational age at birth, birthweight, ethnicity, breastfeeding and daycare attendance. [1]Additionally, models were adjusted for maternal psychological distress. *p-value <0.05, **p-value <0.01

Supplementary Table S4. Association of maternal psychological distress with lung function and asthma at age 10 years, adjusted for different groups of confounders.

	n	FEV$_1$ Z-score (95% CI) n = 3,757	FVC Z-score (95% CI) n = 3,757	FEV$_1$/FVC Z-score (95% CI) n = 3,757	FEF$_{75}$ Z-score (95% CI) n = 3,757	Current asthma OR (95% CI) n = 3,640
**Lifestyle and health-related factors[1]**						
**Overall psychological distress**	4,231					
Per 1-unit increase		-0.09 (-0.19, 0.00)	**-0.11 (-0.20, 0.01)***	0.01 (-0.09, 0.11)	-0.01 (-0.11, 0.08)	**2.01 (1.45, 2.80)****
Clinical cut-off		-0.05 (-0.17, 0.06)	-0.07 (-0.17, 0.04)	0.00 (-0.11, 0.11)	0.01 (-0.10, 0.12)	**2.12 (1.41, 3.19)****
**Depressive symptoms**	4,225					
Per 1-unit increase		-0.06 (-0.12, 0.02)	-0.07 (-0.14, 0.00)	0.01 (-0.06, 0.09)	0.00 (-0.07, 0.07)	**1.53 (1.19, 1.98)****
Clinical cut-off		**-0.14 (-0.26, -0.03)***	**-0.13 (-0.24, -0.02)***	-0.23 (-0.14, 0.09)	-0.05 (-0.15, 0.06)	**2.04 (1.35, 3.08)****
**Anxiety symptoms**	4,226					
Per 1-unit increase		-0.03 (-0.11, 0.05)	-0.03 (-0.10, 0.04)	-0.01 (-0.08, 0.07)	-0.02 (-0.09, 0.06)	**1.67 (1.29, 2.17)****
Clinical cut-off		-0.03 (-0.14, 0.08)	-0.00 (-0.10, 0.10)	-0.07 (-0.17, 0.04)	-0.07 (-0.17, 0.04)	**1.76 (1.18, 2.63)****
**Socio-economic factors[2]**						
**Overall psychological distress**	4,231					
Per 1-unit increase		0.06 (-0.04, 0.16)	0.01 (-0.08, 0.04)	0.09 (-0.00, 0.18)	**0.16 (-0.07, 0.25)****	**2.18 (1.59, 2.99)****
Clinical cut-off		0.10 (-0.01, 0.22)	0.05 (-0.06, 0.16)	0.08 (-0.04, 0.19)	**0.18 (0.07, 0.29)****	**2.42 (1.63, 3.58)****
**Depressive symptoms**	4,225					
Per 1-unit increase		-0.02 (-0.09, 0.06)	0.01 (-0.06, 0.08)	0.07 (-0.00, 0.14)	**0.12 (0.05, 0.19)****	**1.66 (1.30, 2.13)****
Clinical cut-off		-0.01 (-0.13, 0.10)	-0.04 (-0.15, 0.07)	0.05 (-0.07, 0.16)	0.10 (-0.01, 0.21)	**2.26 (1.51, 3.39)****
**Anxiety symptoms**	4,226					
Per 1-unit increase		0.06 (-0.02, 0.14)	0.04 (-0.04, 0.11)	0.03 (-0.04, 0.11)	**0.08 (0.09, 0.15)****	**1.74 (1.35, 2.24)****
Clinical cut-off		0.07 (-0.04, 0.18)	0.08 (-0.03, 0.18)	-0.02 (-0.12, 0.09)	0.05 (-0.05, 0.15)	**1.96 (1.32, 2.91)****
**Birth and early childhood factors[3]**						
**Overall psychological distress**	4,231					
Per 1-unit increase		0.08 (-0.02, 0.17)	0.02 (-0.07, 0.12)	0.09 (-0.00, 0.19)	**0.16, 0.07, 0.25)***	**2.32 (1.69, 3.19)****
Clinical cut-off		**0.12 (0.00, 0.23)***	0.06 (-0.05, 0.17)	0.08 (-0.03, 0.19)	**0.18 (0.07, 0.29)****	**2.56 (1.73, 3.81)****

van Meel ER, *et al. Thorax* 2020; 75:1074–1081. doi: 10.1136/thoraxjnl-2019-214099

**Depressive symptoms**	4,225					
Per 1-unit increase		0.06 (-0.12, 0.33)	0.02 (-0.05, 0.09)	0.07 (-0.01, 0.14)	**0.12 (0.05, 0.19)***	**1.72 (1.35, 2.19)****
Clinical cut-off		-0.00 (-0.12, 0.11)	-0.03 (-0.14, 0.08)	0.05 (-0.07, 0.16)	-0.10 (-0.01, 0.21)	**2.39 1.59, 3.57)****
**Anxiety symptoms**	4,226					
Per 1-unit increase		0.07 (-0.00, 0.28)	0.05 (-0.02, 0.12)	0.04 (-0.02, 0.11)	**0.08 (0.01, 0.15)***	**1.84 (1.42, 2.37)****
Clinical cut-off		0.09 (-0.02, 0.20)	0.09 (-0.01, 0.20)	-0.02 (-0.12, 0.09)	0.05 (-0.05,0.15)	**2.06 (1.39, 3.06)****

Values are Z-scores or odds ratios (OR) with 95% confidence interval (95% CI) from linear or logistic regression models, respectively. Maternal psychological distress is treated as continuous variables (per 1-unit increase) or dichotomous variables based on clinical cut-offs (no; yes, where 'no' was the reference category). Forced Expiratory Flow in 1 second (FEV_1), Forced Vital Capacity (FVC), Forced Expiratory Flow after exhaling 95% of FVC (FEF_{75}). The models were adjusted for [1]smoking during pregnancy, body mass index at enrolment and history of asthma or atopy, [2]maternal age, parity, education level and pet keeping, and child's sex and ethnicity, and [3]child's gestational age at birth, birthweight, breastfeeding and daycare attendance. *p-value <0.05, **p-value <0.01.

Supplementary Table S5. Associations of paternal psychological distress during pregnancy with lung function and asthma at age of 10 years adjusted for paternal psychological distress at 36 months after pregnancy.

	n	FEV_1 Z-score (95% CI) n = 3,757	FVC Z-score (95% CI) n = 3,757	FEV_1/FVC Z-score (95% CI) n = 3,757	FEF_{75} Z-score (95% CI) n = 3,757	Current asthma OR (95% CI) n = 3,640
**Paternal psychological distress during pregnancy + at 36 mo**						
**Depressive symptoms**	4.231					
Per 1-unit increase		-0.04 (-0.20, 0.12)	-0.07 (-0.22, 0.09)	0.04 (-0.09, 0.16)	0.01 (-0.15, 0.16)	1.04 (0.56, 1.93)
Clinical cut-off		-0.10 (-0.33, 0.14)	-0.10 (-0.33, 0.13)	-0.01 (-0.20, 0.17)	-0.06 (-0.25, 0.14)	1.07 (0.43, 2.65)
**Anxiety symptoms**	4,231					
Per 1-unit increase		-0.03 (-0.19, 0.13)	-0.06 (-0.21, 0.10)	0.03 (-0.10, 0.17)	-0.01 (-0.16, 0.14)	0.81 (0.43, 1.55)
Clinical cut-off		**-0.15 (-0.29, -0.00)***	**-0.14 (-0.29, -0.00)***	0.00 (-0.15, 0.15)	-0.07 (-0.22, 0.08)	0.94 (0.48, 1.84)

Values are Z-scores or odds ratios (OR) with 95% confidence interval (95% CI) from linear or logistic regression models, respectively. Maternal psychological distress are treated as continuous variables (per 1-unit increase) or dichotomous variables based on clinical cut-offs (no; yes, where 'no' was the reference category). Forced Expiratory Flow in 1 second (FEV_1), Forced Vital Capacity (FVC), Forced Expiratory Flow after exhaling 95% of FVC (FEF_{75}). The models were adjusted for maternal age, parity, education level, smoking during pregnancy, body mass index at enrolment, history of asthma or atopy and pet keeping, and child's sex, gestational age at birth, birthweight, ethnicity, breastfeeding and daycare attendance, and paternal psychological distress 36 months after pregnancy. At 36 months after pregnancy, not all subscales were measured, and therefor overall psychological distress could not be included at this time point. * p-value <0.05

Supplementary Table S6. Percentage change for associations of paternal psychological distress with lung function and asthma at age 10 years, adjusted for paternal psychological distress at 36 months after pregnancy.

	**FEV_1 Z-score (95% CI) n = 3,757**	**FVC Z-score (95% CI) n = 3,757**	**FEV_1/FVC Z-score (95% CI) n = 3,757**	**FEF_{75} Z-score (95% CI) n = 3,757**	**Current asthma OR (95% CI) n = 3,640**
**Main model + paternal psychological distress at 36 mo**					
**Depressive symptoms**					
Per 1-unit increase	9.8 (-366.8, 374.0)	-62.1 (-703.5, 713.1)	52.4 (-508.9, 537.9)	-179.3 (-579.4, 633.6)	-73.0 (-586.6, 619.1)
Clinical cut-off	6.0 (-156.0, 182.5)	7.3 (-158.3, 176.0)	-34.1 (-242.0, 260.7)	-20.0 (-270.3, 258.9)	-55.2 (-316.3, 306.5)
**Anxiety symptoms**					
Per 1-unit increase	201.4 (-581.6, 611.3)	84.2 (-706.2, 619.0)	29.3 (-628.5, 604.8)	35.4 (-635.3, 619.8)	193.1 (-846.1, 758.8)
Clinical cut-off	9.6 (-31.3, 93.5)	10.0 (-28.3, 102.1)	-2.9 (-320.1, 315.8)	9.8 (-231.8, 227.7)	-381.9 (-425.0, 371.1)

Values are percentage change (95% CI) between the main model, and the model additionally adjusted for paternal psychological distress at 36 months after pregnancy. Paternal psychological distress is treated as continuous variables (per 1-unit increase) or dichotomous variables based on clinical cut-offs (no; yes, where 'no' was the reference category). Forced Expiratory Flow in 1 second (FEV_1), Forced Vital Capacity (FVC), Forced Expiratory Flow after exhaling 95% of FVC (FEF_{75}). The main models were adjusted for maternal age, parity, education level, smoking during pregnancy, body mass index at enrolment, history of asthma or atopy and pet keeping, and child's sex, gestational age at birth, birthweight, ethnicity, breastfeeding and daycare attendance. Additionally, models were adjusted for paternal psychological distress at 36 months after pregnancy. At 36 months after pregnancy, not all subscales were measured, and therefor overall psychological distress could not be included at this time point.

Supplementary Figure S1. Flowchart of participants included for analysis.

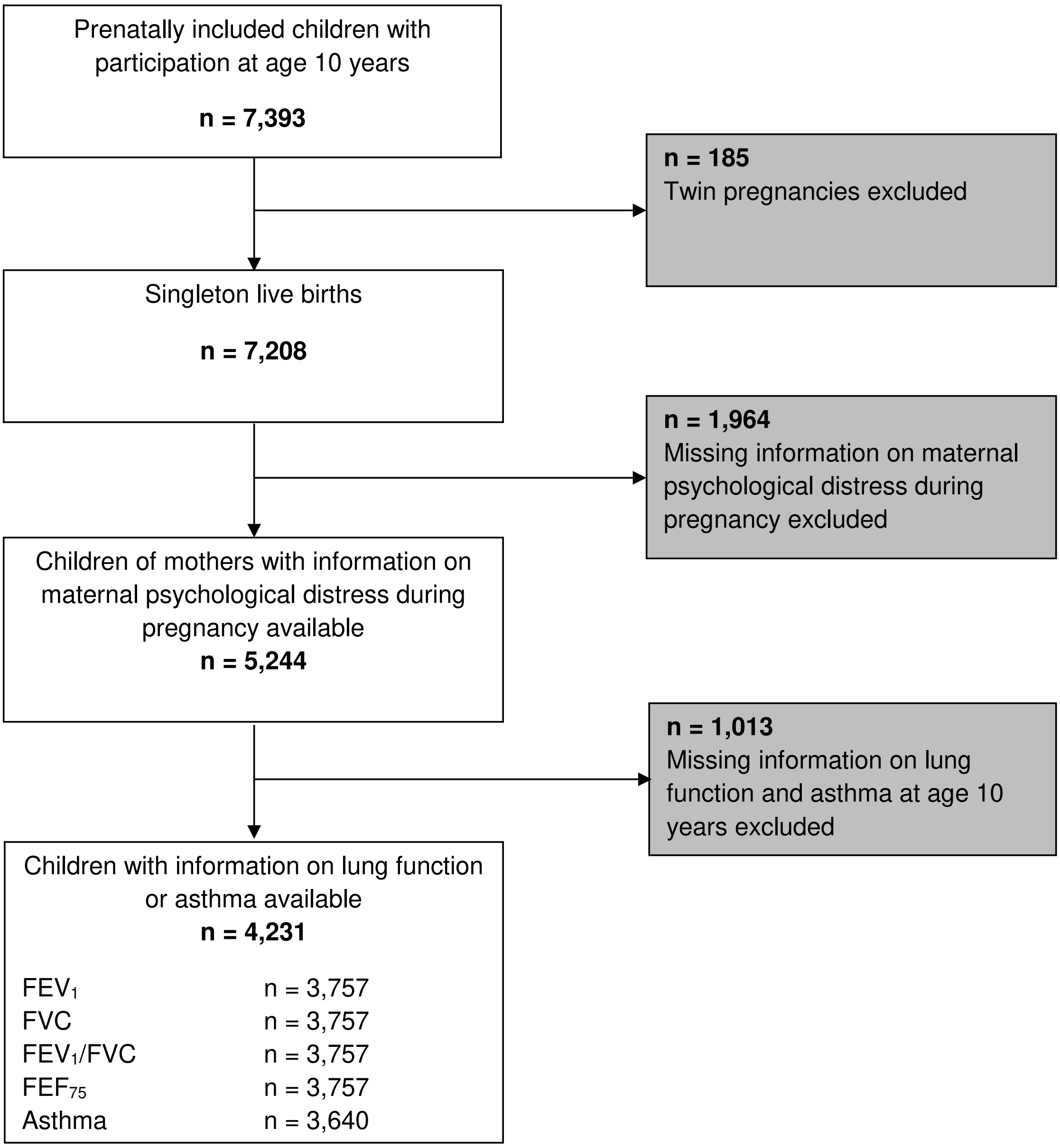

Supplementary Figure S2. Directed acyclic graph

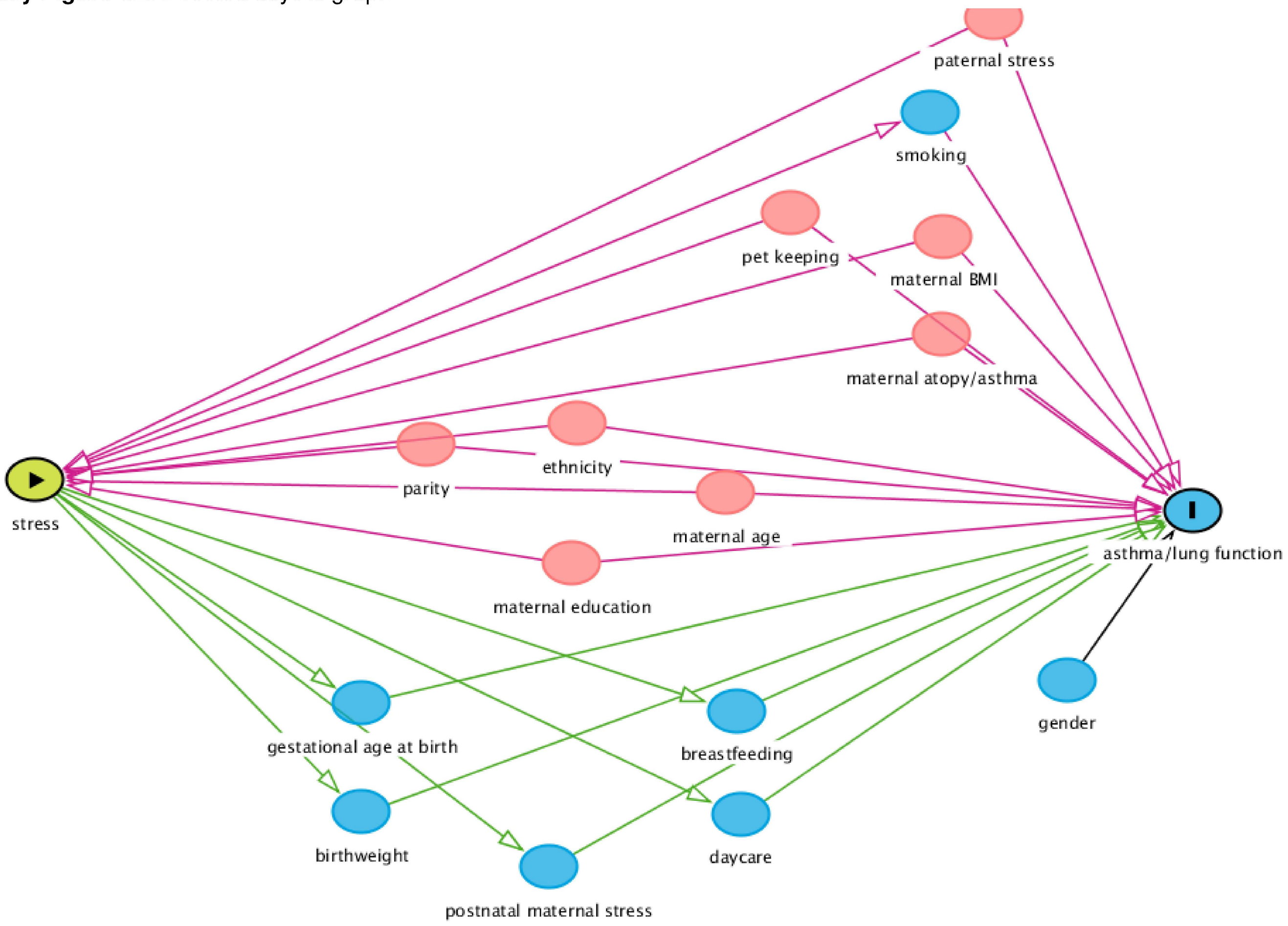

Supplement: Supplementary data [file thoraxjnl-2019-214099supp001.pdf]
